# Pharmacy Students’ Attitudes and Perceptions toward Financial Management Education

**DOI:** 10.3390/healthcare10040683

**Published:** 2022-04-05

**Authors:** Georges Adunlin, Kevin Pan

**Affiliations:** 1 Department of Pharmaceutical, Social and Administrative Sciences, McWhorter School of Pharmacy, Samford University, 800 Lakeshore Drive, Birmingham, AL 35229, USA; 2 Department of Economics, Finance and Quantitative Analysis, Brock School of Business, Samford University, 800 Lakeshore Drive, Birmingham, AL 35229, USA; kpan@samford.edu

**Keywords:** financial management, pharmacy management, business, entrepreneurship, pharmacy students, perception, attitudes, ability

## Abstract

(1) Background: Pharmacy-related financial management training and education are an integral part of the pharmacy curriculum. This study aims to evaluate pharmacy students’ perceptions toward financial management education, their attitudes on its clinical relevance, and their ability to use financial management knowledge in introductory and advanced pharmacy practice experiences. (2) Methods: An online survey was sent to third- and fourth-year pharmacy students. The survey assessed the following three themes: perceptions toward financial management education; attitudes toward the clinical relevance of financial management education; and the student’s ability to use knowledge of financial management in practice. Descriptive statistics were used to summarize the data. (3) Results: The overall response rate for the survey was 60% (139/233). Overall, the study showed a positive perception and attitude toward financial management education. Results indicate that pharmacy students were confident in their ability to use financial management knowledge in pharmacy practice. (4) Conclusions: This survey found an overall optimism in financial management education’s role in pharmacy practice and the ability to obtain financial management competencies in professional pharmacy training. With the evolving practice requirements, pharmacy schools should adapt their financial management curricula with relevant skills to prepare students to become effective entrepreneurs, innovators, and practice leaders.

## 1. Introduction

Financial management plays an important role in every business enterprise ranging from manufacturing, logistics, to healthcare [1]. Without funding and proper planning, organizing, directing, and controlling of its financial activities, a healthcare organization would not be profitable, grow, or likely survive [1]. In today’s rapidly changing healthcare environment, financial management plays a critical role in helping providers and institutions to identify new sources of revenue, find innovative ways to reduce spending and manage long-term investments. Other key aspects of financial management in healthcare include managing contracts to prevent costly mistakes and ensuring regulatory compliance, establishing sound risk-management strategies related to patient safety, and securing sufficient day-to-day financing [1].

In recent decades, the role of pharmacists in the United States has evolved along with the healthcare needs of the population [2,3,4]. The role of pharmacists has extended beyond medication distribution to screenings and consultations [5,6]. In addition to dispensing medications and ensuring patient safety, today’s pharmacists must deliver a range of progressive profit-driven services [7,8], leading them to take on a more significant managerial and entrepreneurial role [9]. The pharmacist is also being given more responsibility in patient care, such as in vaccination services [10,11]. With the release of the Center for Advancement of Pharmacy Education (CAPE) Educational Outcomes 2013 [12], and the National Association of Boards of Pharmacy Curriculum Outcomes Assessment (PCOA) content areas and sub-areas [13], the essentials for practice and care and pharmacy practice management have received a new emphasis in pharmacy education. The CAPE subdomain 2.2 addresses financial management and emphasizes medication-use systems management, (Manager)-Manage patient healthcare needs using human, financial, technological, and physical resources to optimize the safety and efficacy of medication use systems [14]. Within the PCOA Social/Behavioral/Administrative Sciences content areas for the 2016–2017 administration, the specific topics related to financial management include economic and humanistic outcomes of healthcare delivery (Section 3.3) and pharmacy practice management (Section 3.4). The Accreditation Council for Pharmacy Education (ACPE) standard broadly addresses aspects of financial management under practice management and underlines the application of sound management principles (including operations, information, resource, fiscal, and personnel) and quality metrics to advance patient care and service delivery within and between various practice settings.

With the rapid change in the healthcare landscape, opportunities for expanding and implementing new services and programs, and the high costs of pharmaceuticals, it is crucial to prepare pharmacy students with literacy in business, management, and finance-related topics relevant to their practices [15,16]. In the context of pharmacy, financial management is commonly associated with independent pharmacy ownership. However, financial management and its associated skills are important in developing clinical pharmacy services in a wide range of practice settings. It could be argued that financial-management competency should be one of the most fundamental of all skills for pharmacists, since all problems faced by pharmacy organizations and their solutions relate to questions related to how to manage financial resources [17,18,19]. However, the topic occupies an uncertain place within pharmacy programs. It does not enjoy the same breadth of course offerings.

In some schools or colleges of pharmacy, the number of credit hours spent on financial management-related topics is unclear because financial management education is not taught as a separate course. Instead, financial management is incorporated into other courses. There is also a divergence in the content of financial management education, the primary cause of which is perhaps that “financial management” has no singular definition, especially within pharmacy programs [20]. There is a need to confirm whether financial management education and training that pharmacy students are receiving generates a good perception, nurtures positive attitudes, and delivers satisfactory competencies in core areas of financial management. Therefore, this study sought to evaluate third- and fourth-year pharmacy students’ perceptions toward financial management education, their attitudes on its clinical relevance, and their ability to use financial management knowledge in their introductory and advanced pharmacy practice experiences. Perception is the awareness of something which is related to previous knowledge [21]. Perception becomes more skillful with practice and experience, and individual’s perception influences opinion, judgment, and understanding of a situation. Attitude is a learned tendency or readiness to evaluate things or react to some ideas or situations in certain ways, either consciously or unconsciously [22]. In typical educational practice, the terms ‘abilities’ and ‘aptitudes’ are used interchangeably to denote an individual’s potential for acquiring and applying new knowledge or skills [22]. 

## 2. Materials and Methods

### 2.1. Course Description

Financial Management is a required 3-credit-hour semester course in the pharmacy curriculum at Samford University McWhorter School of Pharmacy. It is taught in the Fall semester of the second academic year of didactic coursework. The course is scheduled for weekly 3 h classroom sessions over a 15-week semester calendar. The class meets each week for 2 h on Tuesdays and 1 h on Thursdays. The course catalog description is “Financial Management addresses concepts related to the fiscal management of pharmacy services at the system, pharmacy, and patient-level in various practice settings. Emphasizes decision-making related to the evaluation, procurement, and utilization of financial resources to maximize the value of the organization and to optimize patient care.” The course is organized into three main sections, including an overview of financial management, managing money in pharmacy, and managing pharmacy products and services. The topics covered are listed in Table 1. In the course, all modules are built within the following framework:

- Description and learning objectives;
- PowerPoint lecture;
- Reading and/or listening assignments;
- Reading comprehension questions and/or activities;
- Class discussion questions;
- Additional resources.

**Table 1:** Course Sections and Topics.

Course Section	Topics
Course Section 1: Overview of Financial Management	Management and Management Functions Innovation and Entrepreneurship Strategic Planning to Achieve Results Justifying, Planning, Developing, and Evaluating Clinical Pharmacy Services Risk Management in Contemporary Pharmacy Practice Pharmacy Business & Staff Planning Legal Aspects of Starting and Managing a Pharmacy Business Writing a Pharmacy Business Plan
Course Section 2: Managing Money in Pharmacy	Principles of Accounting Financial Statement Analysis and Ratio Analysis Budgeting Break-even Analysis
Course Section 3: Managing Pharmacy Products and Services	Purchasing and Inventory Management Pricing Pharmacy Products and Services Pharmacy Merchandising Pharmacy Customer Service Marketing Strategies, Advertising, and Promotion Value-Added Services

In addition to didactic lectures, and guest speakers’ presentations, pharmacy students work individually to develop a pharmacy business plan that details a business idea—in this case, a new or expanded pharmacy service or product. This project represents the synthesis, and demonstrates the application, of the knowledge acquired during the course. The students are also presented with case studies and simulation exercises in which they are required to devise strategies and make decisions to ensure the success of a pharmacy organization. Two textbooks are required in this course [23,24]. Journal articles and other readings are assigned for some specific lectures. These practical resources focus on applying knowledge to develop an in-depth understanding of financial management ideas, issues, and concepts.

### 2.2. Study Design, Population, and Samples

A cross-sectional survey was administered to the previous two cohorts enrolled in the course, consisting of third- and fourth-year students. These two cohorts were surveyed with the hypothesis that fourth-year students have a more positive opinion due to their exposure to real-world experience with financial aspects of pharmacy during their advanced pharmacy practice experiences (APPEs), commonly referred to as “rotations”. The third-year students participated in the class in the fall of 2020, while the fourth-year students took the class in the fall of 2019. The total number of students enrolled in the third year was 119, while the number of students enrolled in the fourth year was 114. Therefore, an ideal sample size of 146 participants in total was calculated a priori to achieve an effect size of 0.20, with a power of 0.80 at the alpha level of 0.05 [25,26]. The survey was conducted and managed using Qualtrics XM (Qualtrics, Provo, UT, USA), an online survey-development platform. The survey was delivered via a link through the classes’ mailing lists. To obtain responses that were as truthful as possible, the survey was made anonymous, thus, students were not prompted to provide any identifying information that would reveal their identity. The survey was open for 3 weeks (7–28 November 2021), with two email communications, including the initial survey launch and one reminder.

### 2.3. Survey Instrument

The survey questionnaire was created by modifying various surveys found in the literature [27,28,29]. While most of the survey questions were adapted and modified from previous literature to apply to financial management, a few were developed by the author. A draft version of the survey was distributed to two faculty members within the school of pharmacy in which the study was conducted, and two other faculty members at two other schools of pharmacy to assess its readability and content validity. The survey was also pretested among a group of four randomly selected pharmacy students that were not part of the study population to test clarity, relevance, acceptability, and time to completion (i.e., face validity). Modifications were made as required in terms of language comprehension, font size, and question organization before distributing the final survey to the students. A major modification included consistency with the use of the term ‘pharmacy financial management’ throughout the survey. This was suggested to prevent any confusion and indicate to the student that the survey assessment was strictly based on the instructions received within the course. Another major alteration was made in the ability section of the survey, where each statement was associated with a specific financial management subtopic to facilitate students’ comprehension of these statements.

The final structured survey consisted of a total of 19 questions that could be completed within 5 min. The survey included seven demographic questions and 12 statements divided into three sections asking the students about their level of agreement in terms of their perception attitudes towards financial management education, and their ability to use such knowledge in practice. The participants indicated their level of agreement with the statements using a five-item Likert- type scale. Answers included “strongly disagree”, “disagree”, “neither agree nor disagree”, “agree”, and “strongly agree”.

### 2.4. Data Analysis

Descriptive statistics were used to summarize the data. Incomplete surveys were only included in the analysis if they contained full responses for all the 12 statements on perception, attitude, and ability as well as partial responses to the demographic questions. Therefore, the number of respondents for each question varied. Data were analyzed using SPSS Statistics for Windows, Version 28.01 (IBM SPSS Statistics for Windows, Version 28.0. Armonk, NY, USA: IBM. Corp).

## 3. Results

### 3.1. Demographic Characteristics

Of the 233 students eligible to complete the survey, 139 (60%) students completed the survey, which included 77 third-year students and 62 fourth-year students. The demographic characteristics of respondents are summarized in Table 2.

The demographic characteristics of the third-year students and the fourth-year students are shown in Table 2. To compare the frequency distributions of the two years, a Chi-Square test of independence was used. For gender, third-year students included 72.7% female and fourth-year students included 56.5% female; there was no statistical significance in the difference between third-year and fourth-year (*p*-value = 0.090). For age, most of the third-year respondents (64.9%) were younger than 25 years, while 46.8% of fourth-year respondents were younger than 25 years; there was a statistical significance in the difference between third-year and fourth-year at the significance level of 0.05 (*p*-value = 0.032). This was not surprising, since fourth-year students are expected to be one year older than third-year students. For the highest degree achieved before pharmacy school, there was no statistical significance in the difference between third-year and fourth-year students (*p*-value = 0.321). In terms of prior business courses, 58.7% of third-year and 73.2% of fourth-year had taken business-related courses prior to pharmacy school; therefore, there was no statistical significance in the difference between third-year and fourth-year (*p*-value = 0.156). Lastly, for postgraduate plans, for third-year, 56.6% chose hospital pharmacy, 17.4% chose community pharmacy, 6.5% chose pharmaceutical industry, and 19.6% undecided; for fourth-year, 39.0% chose hospital pharmacy, 36.6% chose community pharmacy, 22.0% chose pharmaceutical industry, and 2.4% were undecided. There is a statistical significance in the difference between third-year and fourth-year students, with a *p*-value of 0.003 at a significance level of 0.01, according to a Chi-square test. For postgraduate plans, since some cells have values less than 5, to test the difference between third-year and fourth-year students, we also applied Fisher’s exact test (IBM SPSS (Armonk, NY, USA: IBM. Corp.)), which can be applied when cell values are less than 5. Fisher’s exact test confirms that there was statistical significance, with a *p*-value of 0.002. The difference between third-yead and fourth-year postgraduate plans was not surprising, since fourth-year students had more experience in clinical rotations than third-year students, and therefore might change their career choices.

### 3.2. Perception of the Clinical Relevance of Pharmacy Financial Management Education

Table 3 shows students’ perception of the clinical relevance of pharmacy financial management education. Four questions were used to assess the perception of the clinical relevance of pharmacy financial management education among the survey respondents. Most respondents agreed that financial management is an integral part of the pharmacy profession (*n* = 66, 46.2%), they may encounter financial management-related questions during their practice as pharmacists (*n* = 63, 44.1%) and that financial management competencies are useful for effective pharmacy practice in today’s health care environment (*n* = 66, 46.2%). More than half of the respondents (*n* = 72, 50.3%) agreed that financial management competencies are useful skills and functions that pharmacists can use to manage aspects of pharmacy operations. The responses were stratified according to the professional year program and by business-related courses received prior to enrolling in pharmacy school (see Appendix A).

### 3.3. Attitudes toward Pharmacy Financial Management Education

Table 4 shows students ’attitudes toward pharmacy financial management education. Most of the respondents agreed that financial management has been a relevant part of their Doctor of Pharmacy curriculum (*n* = 57, 40.1%), financial management should be covered in detail for all colleges and schools of pharmacy (*n* = 58, 40.8%) and that final-year (fourth-year) pharmacy students should be required to have a substantial knowledge of financial management prior to graduation (*n* = 54, 38.0%). The majority also agreed that they intend to read more about financial management, especially in terms of how it influences their practice and/or specialty post-graduation (*n* = 52, 36.6%). The responses were stratified according to the professional year program and business-related courses received prior to enrolling in pharmacy school (see Appendix B).

### 3.4. Ability to Use Pharmacy Financial Management Knowledge in Practice

Table 5 shows students’ the ability to use pharmacy financial management knowledge in practice. Most of the respondents agreed that they were able to manage pharmacy operations (*n* = 64, 46.0%) and manage value-added pharmacy services (*n* = 66, 47.5%). More than half of the respondents agreed that they are able to manage people (*n* = 74, 53.2%), and manage money (*n* = 75, 54.0%). The responses were stratified according to the professional year program and business-related courses received prior to enrolling in pharmacy school (see Appendix C).

## 4. Discussion

This study is one of several that have been conducted in recent years to discuss financial management and business education in pharmacy [15,30,31,32]. However, it is one of the few of its kind used to assess United States pharmacy students’ perceptions and attitudes toward financial management education, and their ability to use their knowledge of financial management in practice. Overall, the study showed a positive perception and attitude toward financial management education. Most of the participants reported being able to use pharmacy financial management in their practice.

Pharmacy students are exposed to financial management and business-related coursework and experiential learning opportunities more than ever before, both within and outside their pharmacy programs [30,33,34,35]. Several trends in pharmacy have influenced this growth, including the expanding role of pharmacists and responsibilities within healthcare organizations, changes in accreditation standards, and educational outcomes that emphasize a wider range of skills relevant to pharmacists. These trends reflect broader economic conditions and shifts in the healthcare system which affect pharmacy practice [36]. Other important factors are changes in pharmacy practice models and patients’ expectations and knowledge, as well as the rapid development of technology in the medication-use process [37,38,39].

In the context of United States pharmacy education, courses and programs that deliver financial management skills, knowledge, and experiences to students are very diverse in terms of key objectives, lecture or credit hours provided, and the professional year in which the financial management course is offered [20]. Given that financial management provides an integrated set of concepts and applications, drawing from entrepreneurship, finance, business, accounting, marketing, and management, pharmacy programs can also vary considerably in terms of their desired outcomes [35,40]. Certain pharmacy programs focus on business management (concentrating on accounting, financial statements, and financial statement analysis) [34,41], while others focus on entrepreneurship, innovation, and creativity to develop new opportunities for pharmacists [20]. On a more pragmatic level, program requirements diverge. Several programs emphasize experiential learning and extra-curricular activities that may or may not be tied to a specific course and credit hours, while others involve a specific sequence of courses for credit [42]. These experiential learning and extra-curricular activities are designed to expose pharmacy students to real business by means of company and pharmacy visits, teaching cooperation, practical training, and providing entrepreneurship-in-residence programs. The entrepreneurship-in-residence programs typically provide pharmacy students with opportunities to engage with accomplished entrepreneurs from the business community. The coaching sessions offered by those entrepreneurs in residence allow pharmacy students to learn about the business environment, beyond the formal curriculum and classroom setting. Even though some programs provide a few hours of instructions on financial management as part of a required course, they do however offer in-depth instruction on financial management as part of an elective course [33,43,44]. Some features distinguish financial management in pharmacy education from other pharmacy courses and influence its structure, emphasis, and outcomes. In many instances, business, finance, and management-related topics are not part of the prerequisite academic work required for entry into pharmacy programs at several institutions. Second, the business environment in which a pharmacy program operates can also play an important role in the ability to leverage important resources to develop a comprehensive and engaging financial management course.

The lesson learned while undertaking this work call for a redesign of the financial management course to include an experiential component. In the previous financial management course structure, students learned a great deal about financial management and acquired a lot of information, but were not provided exposure and hands-on experience outside of the classroom. To overcome this limitation, two courses have been included in the new pharmacy curriculum ‘Practice- and Team-Ready Curriculum’ to provide access to more hands-on experience. In the new curriculum, the Financial Management course is offered in the second year of the program, and a Management, Innovation, Leadership, and Entrepreneurship (MILE) course is offered in the fourth year. These courses are designed to advance pharmacy students who develop entrepreneurial skills in both didactic and experiential work. The MILE course aims to provide pharmacy students with management, innovation, leadership, intrapreneurial, and entrepreneurial knowledge, tools, and skills to allow them to participate effectively in the creation and growth of high-impact pharmaceutical business ventures. Students will have an opportunity to develop their ideas in a team-based setting, identify needs, assess opportunities, and cultivate a lasting competitive advantage when creating innovative products and services with the potential for implementation/commercialization.

Overall, the aim of this work was not to draw representative conclusions regarding all United States pharmacy students, but instead to understand how pharmacy students’ perspective of financial management education could inform curriculum development. Moreover, it was not the immediate purpose of this study to be prescriptive about course content in financial management, as the findings provide information about student respondents’ perception and attitude toward the financial management course taught in their schools of pharmacy. This information can be useful to curriculum committees interesting in making changes to their curriculum. Having knowledge of what other schools or colleges of pharmacy cover in their financial management courses may have some utility. As a next step, we will assess the breadth, depth, and perceived importance of financial management instruction and the level of faculty development in this area in schools and colleges of pharmacy in the United States.

Certain limitations of this study should be considered in the interpretation of the results, their generalization to other educational contexts, and comparison with other studies. The survey responses were conducted from a sample consisting of student pharmacists at one academic institution, which may limit its generalizability and may influence study findings. The survey was dependent upon voluntary subject participation which made it particularly vulnerable to sampling bias. Because of the cross-sectional nature of the study, there is a possibility of self-report bias. While student respondents were asked about experiences that would have taken place within a relatively recent period, recall bias may have occurred. This issue should be addressed in future work using other study designs including using quasi-experimental or repeated measure designs. Since this study was not longitudinal, it would be presumptuous to draw conclusions about changes in students’ perceptions, attitudes, and abilities over time.

## 5. Conclusions

This study has clear educational implications. With ever-increasing pressure to reduce healthcare spending and improve patient outcomes, the need for pharmacists skilled in both the clinical and business aspects of pharmacy is warranted. With so many pharmacy career pathways that require business skills and the growing interest in entrepreneurship, pharmacy students need to be offered the opportunity to access essential financial management training without adding additional time to their degree. Regardless of how pharmacy programs incorporate financial management, curricula must remain dynamic and respond to changes in the healthcare landscape. Future studies should clarify which teaching strategies are suitable for financial management education, as well as the amount of financial management education that is best suited to achieve competency in the pharmacy field.

## Figures and Tables

**Table 2:** Demographic characteristics of Third- and fourth-year Pharmacy Student Respondents.

Characteristics	Overall (*n* = 139) Frequency (Percentage)	Third-Year (*n* = 77) Frequency (Percentage)	Fourth-Year (*n* = 62) Frequency (Percentage)	Chi-Square *p*-Value of Third-Year vs. Fourth-Year
Gender	(*n* = 139)	(*n* = 77)	(*n* = 62)	
Female Male Prefer not to answer	91 (65.5) 47 (33.8) 1 (0.7)	56 (72.7) 21 (27.3) 0 (0)	35 (56.5) 26 (41.9) 1 (1.6)	0.090
Age	(*n* = 139)	(*n* = 77)	(*n* = 62)	
<25 years old ≥25 years	79 (56.8) 60 (43.4)	50 (64.9) 27 (35.1)	29 (46.8) 33 (53.2)	0.032*
Highest degree achieved before pharmacy school	(*n* = 139)	(*n* = 77)	(*n* = 62)	
High school diploma Associate degree Bachelor’s degree Master’s degree	47 (33.8) 16 (11.5) 67 (48.2) 9 (6.5)	22 (28.6) 11 (14.3) 40 (51.9) 4 (5.2)	25 (40.3) 5 (8.1) 27 (43.5) 5 (8.1)	0.321
Taken business-related courses prior to pharmacy school	(*n* = 87)	(*n* = 46)	(*n* = 41)	
Yes No	57 (65.5) 30 (34.5)	27 (58.7) 19 (41.3)	30 (73.2) 11 (26.8)	0.156
Postgraduate plans	(*n* = 87)	(*n* = 46)	(*n* = 41)	
Hospital pharmacy Community pharmacy Pharmaceutical industry Undecided	42 (48.3) 23 (26.4) 12 (13.8) 10 (11.5)	26 (56.5) 8 (17.4) 3 (6.5) 9 (19.6)	16 (39.0) 15 (36.6) 9 (22.0) 1 (2.4)	0.003 ** (Fisher’s exact test *p*-value = 0.002 **) ^+^

* *p*-value < 0.05; ** *p*-value < 0.01; ^+^ For postgraduate plans, since some cells have values less than 5, to test the difference between third-year and fourth-year, we also applied Fisher’s exact test (IBM SPSS) which evaluates the statistical significance of the difference between third-year and fourth-year.

**Table 3:** Perception of the clinical relevance of pharmacy financial management education (*n* = 139).

Statement	Strongly Agree *n* (%)	Agree *n* (%)	Neither Agree nor Disagree *n* (%)	Disagree *n* (%)	Strongly Disagree *n* (%)
Financial management is an integral part of the pharmacy profession.	47 (32.9)	66 (46.2)	15 (10.5)	12 (8.4)	3 (2.1)
I may encounter financial management-related questions during my practice as a pharmacist.	41 (28.7)	63 (44.1)	20 (14.0)	15 (10.5)	4 (2.8)
Financial management competencies are useful for effective pharmacy practice in today’s health care environment.	42 (29.4)	66 (46.2)	16 (11.2)	15 (10.5)	4 (2.8)
Financial management competencies are useful skills and functions that pharmacists can use to manage aspects of pharmacy operations using appropriate data and procedures and/or improve clinical processes and patient care.	47 (32.9)	72 (50.3)	8 (5.6)	13 (9.1)	3 (2.1)

**Table 4:** Attitudes toward pharmacy financial management education (*n* = 139).

Statement	Strongly Agree *n* (%)	Agree *n* (%)	Neither Agree nor Disagree *n* (%)	Disagree *n* (%)	Strongly Disagree *n* (%)
Financial management has been a relevant part of my Doctor of Pharmacy curriculum.	28 (19.7)	57 (40.1)	23 (16.2)	22 (15.5)	12 (8.5)
Financial management should be covered in detail for all colleges and schools of pharmacy.	38 (26.8)	58 (40.8)	22 (15.5)	18 (12.7)	6 (4.2)
Final-year (P4) pharmacy students should be required to have substantial knowledge of financial management prior to graduation.	27 (19.0)	54 (38.0)	26 (18.3)	28 (19.7)	7 (4.9)
Post-graduation, I intend to read more about financial management, especially about how it influences my practice and/or specialty.	34 (23.9)	52 (36.6)	16 (11.3)	28 (19.7)	12 (8.5)

**Table 5:** Ability to use pharmacy financial management knowledge in practice (*n* = 139).

Statement	Strongly Agree *n* (%)	Agree *n* (%)	Neither Agree nor Disagree *n* (%)	Disagree *n* (%)	Strongly Disagree *n* (%)
Managing operations: I am able to apply management knowledge related to strategic planning, business planning, operations management, quality, and risk management in typical situations within a pharmacy organization.	28 (20.1)	64 (46.0)	2 (17.3)	18 (12.9)	5 (3.6)
Managing people: I am able to apply management knowledge related to organizational structure and behavior, human resources management functions, performance appraisal systems, and leadership.	28 (20.1)	74 (53.2)	17 (12.2)	16 (11.5)	4 (2.9)
Managing money: I am aware of the underlying principles that guide budgetary and financial management within a pharmacy organization.	28 (20.1)	75 (54.0)	17 (12.2)	15 (10.8)	4 (2.9)
Managing value-added services: I am able to apply management knowledge related to evaluating the market for and implementing value-added pharmacy services.	29 (20.9)	66 (47.5)	23 (16.5)	17 (12.2)	4 (2.9)

## Data Availability

The data presented in this study are available upon request from the corresponding author.

## Appendix A. Perception Tables

**Table A1:** Perception of the clinical relevance of pharmacy financial management education by professional pharmacy year (N = 139).

Statement	Strongly Agree		Agree		Neither Agree nor Disagree		Disagree		Strongly Disagree	
	3rd Year	4th Year	3rd Year	4th Year	3rd Year	4th Year	3rd Year	4th Year	3rd Year	4th Year
Financial management is an integral part of the pharmacy profession.	25 (32.47)	30 (32.26)	40 (51.95)	26 (41.94)	9 (11.69)	5 (8.06)	2 (2.60)	9 (14.52)	1 (1.30)	2 (3.23)
I may encounter financial management-related questions during my practice as a pharmacist.	24 (31.17)	16 (25.81)	37 (48.05)	25 (40.32)	9 (11.69)	10 (16.13)	5 (6.49)	9 (14.52)	2 (2.60)	2 (3.23)
Financial management competencies are useful for effective pharmacy practice in today’s health care environment.	23 (29.87)	18 (29.03)	43 (55.84)	22 (35.48)	5 (6.49)	10 (16.13)	5 (6.49)	9 (14.52)	1 (1.30)	3 (4.84)
Financial management competencies are useful skills and functions that pharmacists can use to manage aspects of pharmacy operations using appropriate data and procedures and/or improve clinical processes and patient care.	26 (33.77)	20 (32.26)	42 (54.55)	29 (46.77)	3 (3.90)	4 (6.45)	5 (6.49)	7 (11.29)	1 (1.30)	2 (3.23)

**Table A2:** Perception of the clinical relevance of pharmacy financial management education (N = 139) by business related courses prior to pharmacy school (N = 139).

Statement	Strongly Agree		Agree		Neither Agree nor Disagree		Disagree		Strongly Disagree	
	Yes	No	Yes	No	Yes	No	Yes	No	Yes	No
Financial management is an integral part of the pharmacy profession.	23 (40.35)	3 (10.00)	22 (38.60)	20 (66.67)	5 (8.77)	4 (13.33)	6 (10.53)	2 (6.67)	1 (1.75)	1 (1.33)
I may encounter financial management-related questions during my practice as a pharmacist.	20 (35.09)	4 (13.33)	23 (40.35)	17 (56.67)	6 (10.53)	4 (13.33)	8 (14.04)	3 (10.00)	0 (0.00)	2 (6.67)
Financial management competencies are useful for effective pharmacy practice in today’s health care environment.	20 (35.09)	7 (23.33)	25 (43.86)	14 (46.67)	4 (7.02)	4 (13.33)	7 (12.28)	3 (10.00)	1 (1.75)	2 (6.67)
Financial management competencies are useful skills and functions that pharmacists can use to manage aspects of pharmacy operations using appropriate data and procedures and/or improve clinical processes and patient care.	21 (36.84)	6 (20.00)	28 (49.12)	19 (63.33)	2 (3.51)	1 (3.33)	5 (8.77)	2 (6.67)	1 (1.75)	2 (6.67)

## Appendix B. Attitudes Tables

**Table A3:** Attitudes toward pharmacy financial management education by professional pharmacy year (N = 139).

Statement	Strongly Agree		Agree		Neither Agree nor Disagree		Disagree		Strongly Disagree	
	3rd Year	4th Year	3rd Year	4th Year	3rd Year	4th Year	3rd Year	4th Year	3rd Year	4th Year
Financial management has been a relevant part of my Doctor of Pharmacy curriculum.	16 (20.78)	12 (19.35)	32 (41.56)	24 (38.71)	15 (19.48)	7 (11.29)	9 (11.69)	12 (19.35)	5 (6.49)	7 (11.29)
Financial management should be covered in detail for all colleges and schools of pharmacy.	22 (28.57)	15 (24.19)	35 (45.45)	23 (37.10)	11 (14.29)	10 (16.13)	6 (7.79)	11 (17.74)	3 (3.90)	3 (4.84)
Final-year (P4) pharmacy students should be required to have substantial knowledge of financial management prior to graduation.	16 (20.78)	11 (17.14)	29 (37.66)	24 (38.71)	16 (20.78)	9 (14.52)	13 (16.88)	14 (22.58)	3 (3.90)	4 (6.45)
Post-graduation, I intend to read more about financial management, especially about how it influences my practice and/or specialty.	19 (24.68)	15 (24.19)	29 (37.66)	22 (35.48)	9 (11.69)	6 (9.68)	13 (16.88)	14 (22.58)	7 (9.09)	5 (8.06)

**Table A4:** Attitudes toward pharmacy financial management education by business related courses prior to pharmacy school (N = 139).

Statement	Strongly Agree		Agree		Neither Agree nor Disagree		Disagree		Strongly Disagree	
	Yes	No	Yes	No	Yes	No	Yes	No	Yes	No
Financial management has been a relevant part of my Doctor of Pharmacy curriculum.	15 (26.32)	1 (3.33)	23 (40.35)	13 (43.33)	7 (12.28)	6 (20.00)	9 (15.79)	4 (13.33)	3 (5.26)	6 (20.00)
Financial management should be covered in detail for all colleges and schools of pharmacy.	20 (35.09)	3 (10.00)	23 (40.35)	13 (43.33)	4 (7.02)	7 (23.33)	9 (15.79)	3 (10.00)	1 (1.75)	4 (13.33)
Final-year (P4) pharmacy students should be required to have substantial knowledge of financial management prior to graduation.	18 (31.58)	1 (3.33)	19 (33.33)	11 (36.67)	11 (19.30)	7 (23.33)	7 (12.28)	8 (26.67)	2 (3.51)	3 (10.00)
Post-graduation, I intend to read more about financial management, especially about how it influences my practice and/or specialty.	19 (33.33)	4 (13.33)	20 (35.09)	11 (36.67)	6 (10.53)	5 (16.67)	10 (17.54)	4 (13.33)	2 (3.51)	6 (20.00)

## Appendix C. Ability Tables

**Table A5:** Ability to use pharmacy financial management knowledge in practice by professional pharmacy year (N = 139).

Statement	Strongly Agree		Agree		Neither Agree nor Disagree		Disagree		Strongly Disagree	
	3rd Year	4th Year	3rd Year	4th Year	3rd Year	4th Year	3rd Year	4th Year	3rd Year	4th Year
Managing operations: I am able to apply management knowledge related to strategic planning, business planning, operations management, quality, and risk management in typical situations within a pharmacy organization.	17 (17 (22.08)	11 (17.74)	37 (48.05)	27 (43.55)	12 (15.58)	12 (19.35)	9 (11.69)	9 (14.52)	2 (2.60)	3 (4.84)
Managing people: I am able to apply management knowledge related to organizational structure and behavior, human resources management functions, performance appraisal systems, and leadership.	16 (20.78)	12 (19.35)	43 (55.84)	31 (50.00)	9 (11.69)	8 (12.90)	7 (9.09)	9 (14.52)	2 (2.60)	2 (3.23)
Managing money: I am aware of the underlying principles that guide budgetary and financial management within a pharmacy organization.	16 (20.78)	12 (19.35)	44 (57.14)	31 (50.00)	10 (12.99)	7 (11.29)	5 (6.49)	10 (16.13)	2 (2.60)	2 (3.23)
Managing value-added services: I am able to apply management knowledge related to evaluating the market for and implementing value-added pharmacy services.	16 (20.78)	13 (20.97)	40 (51.95)	26 (41.94)	10 (12.99)	13 (20.97)	8 (10.39)	9 (14.52)	3 (3.90)	1 (1.61)

**Table A6:** Ability to use pharmacy financial management knowledge in practice by business related courses prior to pharmacy school (N = 139).

Statement	Strongly Agree		Agree		Neither Agree nor Disagree		Disagree		Strongly Disagree	
	Yes	No	Yes	No	Yes	No	Yes	No	Yes	No
Managing operations: I am able to apply management knowledge related to strategic planning, business planning, operations management, quality, and risk management in typical situations within a pharmacy organization.	16 (28.07)	1 (3.33)	22 (38.60)	14 (46.67)	11 (19.30)	7 (23.33)	7 (12.28)	7 (23.33)	1 (1.75)	1 (3.33)
Managing people: I am able to apply management knowledge related to organizational structure and behavior, human resources management functions, performance appraisal systems, and leadership.	17 (29.82)	2 (6.67)	26 (45.61)	17 (56.67)	6 (10.53)	6 (20.00)	8 (14.04)	4 (13.33)	0 (0.00)	1 (3.33)
Managing money: I am aware of the underlying principles that guide budgetary and financial management within a pharmacy organization.	17 (29.82)	1 (3.33)	28 (49.12)	19 (63.33)	5 (8.77)	6 (20.00)	7 (12.28)	3 (10.00)	0 (0.00)	1 (3.33)
Managing value-added services: I am able to apply management knowledge related to evaluating the market for and implementing value-added pharmacy services.	17 (29.82)	2 (6.67)	25 (43.86)	15 (50.00)	8 (14.04)	6 (20.00)	6 (10.53)	6 (20.00)	1 (1.75)	1 (3.33)
